# Clinical, Immunological, and Molecular Findings in 57 Patients With Severe Combined Immunodeficiency (SCID) From India

**DOI:** 10.3389/fimmu.2019.00023

**Published:** 2019-02-04

**Authors:** Jahnavi Aluri, Mukesh Desai, Maya Gupta, Aparna Dalvi, Antony Terance, Sergio D. Rosenzweig, Jennifer L. Stoddard, Julie E. Niemela, Vasundhara Tamankar, Snehal Mhatre, Umair Bargir, Manasi Kulkarni, Nitin Shah, Amita Aggarwal, Harsha Prasada Lashkari, Vidya Krishna, Geeta Govindaraj, Manas Kalra, Manisha Madkaikar

**Affiliations:** ^1^Department of Pediatric Immunology and Leukocyte Biology, National Institute of Immunohaematology (ICMR), Mumbai, India; ^2^Division of Immunology, Bai Jerbai Wadia Children's Hospital, Mumbai, India; ^3^Department of Pediatric Pulmonology, G. Kuppuswamy Naidu Memorial Hospital, Coimbatore, India; ^4^Department of Laboratory Medicine, NIH Clinical Center, Bethesda, MD, United States; ^5^Centre for Medical Genetics, Mumbai, India; ^6^Pediatric Hematology-Oncology, P. D. Hinduja National Hospital & Research Center, Mumbai, India; ^7^Sanjay Gandhi Postgraduate Institute of Medical Sciences, Lucknow, India; ^8^Department of Pediatrics, Kasturba Medical College, Mangalore, India; ^9^Department of Pediatrics, Sri Ramachandra Medical College and Research Institute, Chennai, India; ^10^Department of Pediatrics, Institute of Maternal and Child Health, Government Medical College, Kozhikode, India; ^11^Department of Pediatrics Hematology and Oncology, Indraprastha Apollo Hospital, New Delhi, India

**Keywords:** PID, flow cytometry, TREC, sanger sequencing, targeted next generation sequencing

## Abstract

Severe combined immunodeficiency (SCID) represents one of the most severe forms of primary immunodeficiency (PID) disorders characterized by impaired cellular and humoral immune responses. Here, we report the clinical, immunological, and molecular findings in 57 patients diagnosed with SCID from India. Majority of our patients (89%) presented within 6 months of age. The most common clinical manifestations observed were recurrent pneumonia (66%), failure to thrive (60%), chronic diarrhea (35%), gastrointestinal infection (21%), and oral candidiasis (21%). Hematopoietic Stem Cell Transplantation (HSCT) is the only curative therapy available for treating these patients. Four patients underwent HSCT in our cohort but had a poor survival outcome. Lymphopenia (absolute lymphocyte counts/μL <2,500) was noted in 63% of the patients. Based on immunophenotypic pattern, majority of the cases were T^−^B^−^ SCID (39%) followed by T^−^B^+^ SCID (28%). MHC class II deficiency accounted for 10.5% of our patient group. A total of 49 patients were molecularly characterized in this study and 32 novel variants were identified in our cohort. The spectrum of genetic defects in our cohort revealed a wide genetic heterogeneity with the major genetic cause being *RAG1/2* gene defect (*n* = 12) followed by *IL2RG* (*n* = 9) and *JAK3* defects (*n* = 9). Rare forms of SCID like Purine nucleoside phosphorylase (PNP) deficiency, reticular dysgenesis, DNA-Protein Kinase (DNA-PKcs) deficiency, six cases of MHC class II deficiency and two ZAP70 deficiency were also identified in our cohort. Fourteen percent of the defects still remained uncharacterized despite the application of next generation sequencing. With the exception of MHC class II deficiency and ZAP70 deficiency, all SCID patients had extremely low T cell receptor excision (TRECs) (<18 copies/μL).

## Introduction

Severe combined immunodeficiency (SCID) refers to a heterogeneous group of primary immunodeficiency disorders characterized by impaired T lymphocyte development with an effect on the B cell and NK cell number and/or function. SCID pathogenesis involves multiple genes whose defect which leads to abnormal cellular and humoral immune responses. Affected children suffer from recurrent infections, notably infections with opportunistic organisms such as *Pneumocystis jiroveci*, chronic diarrhea, failure to thrive and persistent mucocutaneous candidiasis (1). SCID is not apparent at birth and the presence of maternally derived antibodies provide some protection in the initial few months which further delays the diagnosis. As a consequence, these children also get the routine administration of live vaccines which is known to be contraindicated in SCID patients. Diagnosis of SCID is supported by a low absolute lymphocyte counts, abnormalities in lymphocyte subpopulations, absent/reduced naïve T cell population and recent thymic emigrants, absent T cell receptor excision circles (TRECs) and a low or absent T cell response to mitogens.

The Primary Immune Deficiency Treatment Consortium (PIDTC) classifies patients into Typical SCID with total T cell (CD3) count of <300 cells/microL (2). Depending on the B and NK cell status, patients are further classified as T^−^B^+^NK^−^, T^−^B^+^NK^−^, T^−^B^−^NK^+^, T^−^B^−^NK^−^. Some patients can have T cells (leaky/atypical SCID) which are mostly oligoclonal and are classified as T^+^ or T^++^ SCID. These modify the counts from T^−^B^−^NK^−^ to T^+^B^−^NK^−^,T^−^B^+^NK^−^ to T^+^B^+^NK^−^, from T^−^B^−^NK^+^ to T^+^B^−^NK^+^, or from T^−^B^+^NK^+^ to T^+^B^+^NK^+^ (3).

More than 30 genes are involved in SCID pathogenesis (3). Despite a wide genetic heterogeneity, patients are clinically indistinguishable. Importantly, the clinical features in SCID infants can also be found in patients with human immunodeficiency virus (HIV) infection/acquired immunodeficiency syndrome (AIDS). Hence, it is essential to rule out such secondary causes of lymphopenia by determining the presence of maternal HIV antibodies and measuring the levels of HIV by polymerase chain reaction (PCR).

With the exception of common gamma chain cytokine receptor (IL2RG) deficiency which follows X-linked pattern of inheritance, all the other causes are inherited in an autosomal recessive pattern. There are many SCID cases where the genetic defect is still unknown. In countries with a low rate of consanguinity, approximately 50% of all SCID cases are X-linked (4). Of the AR forms of SCID, 20–30% of all SCID patients are T^−^, B^−^, NK^+^, and approximately half of these patients have mutations in the *RAG1* or *RAG2* genes.

The incidence of SCID was previously reported at approximately 1 in 100,000 but the implementation of TREC assay for Newborn screening of SCID revealed the true incidence of SCID to be 1 in 58,000 live births (95% CI, 1 in 46,000–1 in 80,000) for typical SCID, leaky/atypical SCID, and Omenn syndrome (5). SCID is a fatal disorder and without treatment, death from infection usually occurs within the first 2 years of life. Diagnosis must be made before severe life-threatening infections occur so that the immunity can be restored with enzyme replacement or Hematopoietic Stem Cell Transplantation (HSCT); early transplantation (before 3.5 months of age) can lead to long-term survival (6). Gene therapy is an alternative option available especially for patients with ADA-SCID and X-SCID.

Here, we report the first largest series on the clinical, immunological, and molecular findings in SCID patients (*n* = 57) from India.

## Materials and Methods

### Patients and Samples

Patients (*n* = 57) suspected of Severe combined immunodeficiency (SCID) at National Institute of Immunohaematology (NIIH) between 2013 and 2018 were included in the study. Informed consent for participating in the study was procured from the family members in accordance with the declaration of Helsinki and 3 mL peripheral blood was collected in EDTA, Plain and Heparin vacutainers each. The study was approved by the Institutional Ethics Committee of NIIH.

A clinical proforma was filled for all patients which included the age, consanguinity, family history, clinical parameters like number of infections, site of infections, age of presentation, failure to thrive, diarrhea, presence of any skin rashes, administration of vaccines and post live vaccine complications, presence of dysmorphic features, hepatosplenomegaly, lymphadenopathy.

Prenatal diagnosis (PND) was provided to a total of four affected families. Two families were provided a molecular confirmation of the genetic defect on the chorionic villus sample. Maternal contamination was ruled out by Kleihauer-Betke (KB) staining and analysis of the variable number of tandem repeats (VNTR) using the apolipoprotein B (*ApoB)*, ACTB2, D1S80, and *IgJH* genes. Phenotypic prenatal diagnosis was provided to 2 families on the Fetal cord blood (FB) sample (1–2 mL, <0.5% of expected weight in all cases) as molecular diagnosis was not available at the time of PND. The FB sample was collected at 18 weeks of gestation by ultrasound-guided cordocentesis after procuring informed consent from the parents. The FB sample accepted for analysis had a high MCV value (>110 fL) with narrow and single red cell distribution curve. The testing was performed within 3 h of sampling.

### Immunological Workup

Initial investigations involved a complete blood cell count (CBC) on a Sysmex XS-800i (Sysmex Co., Cobe, Japan) 5-part automated hematological analyzer, lymphocyte subset analysis by flow cytometry using BD Multitest 6-color TBNK reagent followed by acquisition of cells on FACS Aria I; analysis was performed on FACS Diva and FlowJo software (BD Biosciences, San Jose, CA, USA). Serum immunoglobulin levels were estimated by nephelometry (BNProspec, Siemens).

The percentage of naïve and memory T cell subsets on CD4^+^ and CD8^+^ cells was measured by flow cytometry using anti-CD45RA phycoerythrin (PE), anti-CD45RO Phycoerythrin/Cy7 (PE-Cy7) and anti-CD62L allophycocyanin (APC) procured from BD Biosciences, San Jose, CA, USA.

T cell receptor excision circles (TRECs) were measured by an in-house modification of a previously described method (7).

Flow cytometric evaluation of Human Leukocyte antigen- D related (HLA-DR) expression on lymphocytes and monocytes using cell surface markers specific for T cells (anti-CD3 Peridinin-chlorophyll-protein Complex: CY5.5 Conjugate, PerCP-Cy5), B cells (anti-CD19 allophycocyanin [APC]), monocytes (anti-CD14 Phycoerythrin [PE]) and HLA-DR (anti-HLA-DR fluorescein isothiocyanate [FITC]) was performed.

T cell proliferation assay was performed using CellTrace Violet dye (Thermo Fisher Scientific). The PBMCs separated from the heparinized blood samples of the patients was suspended in complete RPMI medium (GIBCO, USA) containing 10% fetal calf serum (GIBCO, USA) and was stained at a density of 106 cells with Cell trace violet (1 μM) for 20 min at 37°C. The cells were aliquoted into 96 well tissue culture plates at a density of 105 cells per well and stimulated with phytohemagglutinin (PHA) (1 μg/ml) and cultured for 72 h. Flow cytometric analysis of T cell functionality was assessed on CD3^+^ and CD69^+^ (activation marker) T cells.

Clonality of the T cell receptor (TCR) was assessed by flow cytometric evaluation of TCR-Vβ repertoire by using the IOTest® Beta Mark.

Flow cytometric evaluation of CD132 expression on B cells was done using cell surface markers specific for B cells (anti-CD19 allophycocyanin [APC]) and CD132 (Phycoerythrin [PE]) procured from Biolegend. Flow cytometric evaluation of CD127 on T cells was done using cell surface markers specific for T cells (anti-CD3 fluorescein isothiocyanate [FITC]) and anti-CD127 allophycocyanin [APC]) procured from BD biosciences.

Phospho-STAT5 analysis was performed on whole blood after IL-2 (10 μg/ml) (Peprotech, NJ, USA) stimulation for 15 min at 37°C. The cells were fixed with BD Lyse Fix and permeabilized with Perm III Buffer. The cells were stained with anti-phospho-STAT5 (p-STAT5) Alexa 488 (Y694, clone 47, BD Biosciences) according to the manufacturer's instructions.

Determination of Adenosine deaminase (ADA) activity on RBC lysate was performed by the Giusti and Galant calorimetry method using a commercially available kit (ADA-MTB kit) from Tulip Diagnostics, India.

Data was presented in terms of median and percentages. One-way analysis of variance (ANOVA) test was used for comparison of >2 groups. Mann-Whitney U-test was used for comparing groups with non-parametric data. The *p*-values less than 0.05 were considered statistically significant. All statistical calculations were done using GraphPad prism (Chicago, IL, USA) version 15 for Microsoft Windows.

### Molecular Investigations

Molecular investigations were done by Sanger sequencing of *IL2RG, ADA, RAG1, RAG2, IL7RA, ZAP70* genes using the standard protocol. Targeted Next Generation sequencing was performed using a custom capture kit by Medgenome Labs Pvt Ltd. India, in samples where molecular diagnosis was not identified by Sanger Sequencing. The libraries were sequenced on Illumina sequencing platform (mean coverage >80 to 100X). The identified mutations were confirmed by Sanger sequencing.

## Results

### Patient Characteristics and Clinical Findings

In this study, a total of 57 SCID patients were diagnosed and followed up. Forty patients (70%) were male. Data on the status of consanguinity was available for 53 cases, from which 19 patients (36%) belonged to consanguineous parents. A positive family history of SCID was recorded in 32 families (56%). The median age at onset and diagnosis of all patients referred with a clinical suspicion of SCID was 60 days (range, 12–304) and 152 days (range, 12–730), respectively. Two patients received a pre-symptomatic diagnosis in view of strong family history.

The most common clinical manifestations were pneumonia (66%), failure to thrive (60%), chronic diarrhea (35%), gastrointestinal infection (21%), oral candidiasis (21%) and BCGiosis (12%). In our cohort, organisms isolated included both gram- negative and gram-positive bacterium: *Staphylococcus aureus* (*n* = 2), *Klebsiella pneumonia* (*n* = 8), *Pseudomonas aeruginosa* (*n* = 4), *Burkholderia* (*n* = 1), *Chryseobacterium* (*n* = 1). Viral organisms isolated included rotavirus (*n* = 1), cytomegalovirus (CMV) (*n* = 2), Rubella (*n* = 1), RSV (*n* = 1), Varicella (*n* = 1). Fungal infections included PCP (*n* = 1). Other features such as Erythematous skin rash was observed in 29% of the cases, Dysmorphism was seen in 8% cases, abscess in 8% and Hepatosplenomegaly in 3% cases. Table 1 presents the clinical findings in our patient cohort.

**Table 1:** Clinical findings in SCID patients.

**Pt. code**	**Genetic defect**	**Sex**	**Age at diagnosis (Days)**	**Age of onset (Days)**	**Family history**	**Consanguinity**	**Pneumonia**	**GI infection**	**Organism isolated (source)**	**Oral candidiasis**	**Chronic diarrhea**	**FTT**	**BCGiosis**	**Skin rash**	**Dysmorphic features**	**Other infections**	**Status**
P1	*PNP*	Female	365	15	+	+	+	–	*Candida* (Blood culture)	+	–	+	–	–	–	Meningitis, delayed motor milestones and involuntary movements	Dead/18 months of age
P2	*RAG1 (OS)*	Female	91	46	–	+	–	–	–	–	+	–	–	+	–	Blephatis, Shedding of eyelashes, ear discharge, discharge from umbilical stump	Dead/4 months of age
P3	*RFXAP*	Male	183	183	+	–	+	–	*Burkholderia cepacia* (Blood culture)	–	–	+	–	+	–	Amoeboid blanching skin rash	Dead/7 months of age
P4	*ADA* (Leaky/atypical)	Male	183	20	+	+	+	–	–	–	–	+	–	–	+	Low set ears, frontal bossing, fused eyebrows	Dead/6 months of age
P5	*ZAP70*	Male	365	61	+	+	+	+	–	–	+	–	+	+	–	NS	Dead/18 months of age
P6	*RFXAP*	Male	91	91	+	–	–	–	–	–	–	–	–	–	–	Pre-symptomatic diagnosis	HSCT/Dead at 7 months
P7	*IL2RG*	Male	61	61	+	–	–	–	–	–	–	–	–	–	–	Umbilical sepsis, fever, urinary tract infection	Dead/5 months of age
P8	*IL2RG*	Male	91	ND	ND	ND	+	ND			ND	ND	ND	ND	ND	NS	Dead/6 months of age
P9	*IL2RG*	Male	91	91	–	–	–	–	*Pseudomonas aeruginosa* (Blood culture)	–	–	–	–	–	–	Multiple deep-seated abscess	Dead/5 months of age
P10	*IL2RG*	Male	152	30	+	–	+	–	*Klebsiella pneumonia* (Blood culture)	+	–	+	–	+	–	Oral thrush, convulsion,	Dead/11 months of age
P11	*ADA*	Male	30	45	+	–	+	–	*Klebsiella* (Blood culture)	–	–	+	ND	+	–	Hypoglycemic convulsions, blisters on face and cheeks and hyperpigmentation	Dead/2 months of age
P12	*JAK3* (Leaky/atypical)	Male	183	122	+	–	+	+	*Staphylococcus aureus* (Blood culture)	–	+	+	+	–	–	Soft hepatosplenomegaly	HSCT/Dead at 12 months
P13	*AK2*	Male	12	12	+	+	+	–	*Candida* (Blood culture)	+	–	–	NI	–	–	NS	HSCT/Dead at 2 months
P14	*ADA*	Female	76	30	+	–	+	+	–	–	+	+	–	–	–	NS	Dead/3 months of age
P15	*JAK3*	Male	61	15	–	–	–	–	–	–	–	–	–	–	–	Recurrent boils, multiple scalp abscess and liver abscess, liver abscess	Dead/6 months of age
P16	*RAG1*	Female	753	365	–	–	–	+	*Candida* (Blood culture)	+	+	–	–	–	–	Oesophagul candidiasis, celiac disease	Alive
P17	*ADA*	Male	152	137	+	–	–	–	–	–	–	–	+	–	–	Humerus crack with severe swelling at site for 3-5, hepatosplenomegaly with soft tissue mass in abdomen	Dead/5 months of age
P18	*RAG2*	male	122	122	–	–	+	+	*Rotavirus* (stool culture)	–	+	+	–	+	–	Atopic dermatitis, Impetigo over back	Dead/6 months of age
P19	*-* (Leaky/atypical)	Male	365	122	–	–	+	+	*Gram+ve Bacilli*	–	+	+	–	–	–	Pallor	Dead/2 months of age
P20	*RFX5*	Female	213	91	–	–	+	–	*Candida* (Bronchoalveolar lavage)	–	–	–	–	–	–	Fever, hepatosplenomegaly, motor development delay	HSCT/Dead at 9 months
P21	*RFXAP*	Male	122	15	+	+	+	+	*Candida albicans* (Bronchoalveolar lavage)	+	+	+	–	+	–	NS	Dead/6 months of age
P22	*ADA*	Male	30	15	+	–	–	–	*Klebsiella pneumonia* (Blood culture)	+	–	+	–	+	–	NS	Dead/1.5 months of age
P23	–	Male	213	46	+	ND	+	+	*ND*	–	+	+	–	–	–	Tachypnea with respiratory distress, acute bronchiolitis	Dead/7 months of age
P24	*RAG2*	Male	213	152	–	–	+		*Klebsiella pneumonia* (Blood culture)	–	–	–	–	–	–	NS	Dead/12 months of age
P25	*PRKDC* (leaky)	Male	61	61	+	+	+	–	*RSV* (ND)	–	–	+	–	–	+	low set ears, retrognathia Developmental delay	Lost to follow up
P26	*RAG1* (Leaky/atypical)	Male	730	91	+	+	–	–	–	–	+	–	–	+	–	Nephrotic range of proteinuria,ITP	Dead/36 months of age
P27	–	Female	91	30	+	–	+	–	*Pseudomonas aeruginosa* (Blood culture)	–	–	–	–	+	–	extensive pyoderma	Dead/6 months of age
P28	–	Male	304	304	–	ND	–	–	*Klebsiella* (Blood culture)*, Candida*	+	–	+	–	–	–	Hyponatremia, septic shock, severe dehydration	Dead/12 months of age
P29	*JAK3*	Female	152	106	–	–	+	–	*AFB* (Blood culture)	+	–	+	+	–	–	NS	Dead/8 months of age
P30	–	female	61	61	–	+	+	–	–	+	–	+	–	–	–	Developmental delay, diffuse cerebral atrophy, microcephaly, scalp seborrhea, oral thrush	Lost to follow up
P31	*IL7RA*	Female	213	61	–	+	+	–	*PCP* (ND)	+	+	–	–	–	–	Convulsions	Dead/8 months of age
P32	*DCLER1C*	Male	183	152	+	–	+	–	*Pseudomonas aeruginosa* (Blood culture)	–	–	–	Not immunized	–	–	Acute respiratory distress	Died/7 months of age
P33	*CIITA*	Male	213	122	–	–	–	–	*Chryseobacterium* (Blood culture)	–	–	–	–	–	–	Acute respiratory distress	Alive
P34	– (Leaky/atypical)	Female	243	213	+	ND	+	–	*Candida* (Blood culture)	+	–	–	ND	–	–	Acute fever, positive for H1N1	Lost to follow up
P35	*RAG2* (Leaky/atypical)	Female	243	91	–	–	+	–	–	–	–	+	ND	–	–	NS	Dead/10 months of age
P36	*JAK3*	Male	183	61	+	+	+	–	–	–	+	+	–	–	+	Triangular face, low set ears, dry skin, hair sparse	Dead/6 months of age
P37	*IL2RG*	Male	91	91	+	–	+	–	–	–	–	+	–	+	–	Recurrent fever, hypopigmented fungal perianal rashes	Dead/4 months of age
P38	*RAG1*	Female	122	61	+	–	–	+	–	–	+	+	–	–	+	- Microcephaly, short philtrum, broad nasal bridge	Lost to follow up
P39	*JAK3* (OS)	Female	122	30	–	–	–	–	*MTB* (Sputum culture)	–	–	–	–	+	–	Culture negative sepsis, cutaneous abscess, rectovaginal fistula	Dead/5 months of age
P40	*JAK3*	Male	152	15	–	–	–	–	*Budding yeast with Pseudohyphae* (ND)	–	+	+	+	+	–	NS	Dead/6 months of age
P41	*IL2RG*	Male	213	122	+	–	+	–	*Disseminated BCG* (ND)	–	–	–	+	–	+	Abscess	Dead/9 months of age
P42	*RAG1*	Male	60	60	+	–	+	+	–	–	+	+	–	–		NS	Dead/3 months of age
P43	*RAG2* (OS)	Male	122	183	+	–	+	–	*Klebsiella pneumonia* (Blood culture)	–	+	+	–	+	–	NS	Dead/6 months of age
P44	*IL2RG* (Leaky)	Male	152	0	–	–	–	–	–	–	–	–	–	–	–	Pre-symptomatic diagnosis	Dead/10 months of age
P45	*IL2RG*	Male	183	106	–	–	+	–	–	–	–	+	–	–	–	persistent cough, hypoxia, and respiratory distress	Dead/8 months of age
P46	*DCLER1C*	Male	183	183	+	–	–	–	–	–	–	+	–	–	–	Fever high grade of unknown origin	Dead/8 months of age
P47	–	Male	18	18	+	–				–				–	–	NS	Alive
P48	*RAG*	Male	27	20	–	–	+	–	*Pseudomonas aeruginosa* (Blood culture)	–	–	–	–	–	–	Pallor, fever, cough	Dead/months of age
P49	*RFXANK*	Female	243	122	+	+	+	–	–	–	–	+	–	–	–	NS	Dead/10 months of age
P50	*ZAP70* (Atypical)	Male	304	304	+	+	+	+	*Cryptosporidium, AFB* (ND)	–	+	+	+	+	–	Bilateral axillary lymphadenopathy, erythematous Urticarial rash	Dead/3 months of age
P51	*RAG1* (OS)	Female	46	5	+	–	–	–	*Staph aureus* (Blood culture)	–	–	+	–	+	–	Erythematous scaly eczema like rash, inguinal lymphadenopathy	Dead/3 months of age
P52	*JAK3*	Male	91	183	–	+	+	+	*Klebsiella pneumonia* (Blood culture)	–	+	+	–	–	–	NS	Alive
P53	*ADA*	Male	61	14	–	+	+	–	*Rubella, HSV* (ND)	–	–	+	–	–	–	Severe sepsis increased respiratory activity	Alive
P54	*JAK3*	Female	304	15	+	+	+	–	*Candida* (Blood culture)	+	ND	+	ND	ND	ND	ND	Dead/11 months of age
P55	*JAK3* (Leaky/atypical)	Male	122	30	–	+	+	–	–	–	+	+	–	–	–	NS	Alive
P56	–	Female	61	61	+	+	+	–	–	–	–	+	–	–	–	NS	Dead/3 months of age
P57	*RAG1*	Male	304	10	–	+	+	–	–	–	+	+	–	+	–	Recurrent fever Septic arthritis, Knee abscess	Dead/10 months of age

*ND, No data; “+,” Present; “–,” Absent; NS, Nothing significant; HSCT, Hematopoietic stem cell transplantation; RSV, respiratory syncytial virus; BCG, Bacillus Calmette–Guérin; HSV, herpes simplex virus; MTB, mycobacterium tuberculosis; IL2RG, Interleukin 2 receptor gamma chain; JAK3, Janus Kinase 3; IL7RA, Interleukin 7 receptor alpha; ADA, Adenosine Deaminase; PNP, Purine nucleoside phosphorylase; AK2, adenylate kinase 2; RAG1/2, Recombination activating gene1/2; DCLER1C, DNA Cross-Link Repair 1C; RFXAP, Regulatory Factor X Associated Protein; RFX5, Regulatory factor 5; RFXANK, Regulatory Factor X Associated Ankyrin Containing Protein; CIITA, Class II Major Histocompatibility Complex Transactivator; ZAP70, Zeta-chain-associated protein kinase 70*.

### Immunological Findings

Based on the Primary Immune Deficiency Treatment Consortium (PIDTC) case definition for SCID, 38 of 57 patients (67%) with absent or severely low T cell counts (<300 cells/μL) were classified as typical SCIDs (2). The remaining patients were classified as Leaky/Atypical SCIDs. Lymphopenia (<2,500 lymphocyte counts/μL) was noted in 36 of 57 (63%) patients including both typical and atypical SCID cases. Of these, the median lymphocyte count/μL was 680 (11–2,403) counts/μL. Classification of patients based on comparison of absolute counts/μL of B and NK cells with age matched reference ranges (8) revealed 14 patients with T^−^B^+^NK^−^ phenotype (25%), 2 patients as T^−^B^+^NK^+^ (3.5%), 10 as T^−^B^−^NK^−^ (17.5%), 12 patients with T^−^B^−^NK^+^ SCID (21%).

7 of 57 (12%) cases were identified with isolated T cell lymphopenia (selective deficiency of CD4^+^ T cells (*n* = 6) or CD8^+^ T cells (*n* = 1) and were eventually grouped under the category “Combined Immunodeficiency (CID) generally less profound than Severe combined Immunodeficiency” according to the IUIS classification (9).

The remaining cases with detectable T cells were classified as leaky/atypical SCID (12 cases, 21%). Of these cases, 6 patients had T^+^B^+^NK^−^ phenotype (1 Omenn phenotype), 2 patients as T^+^B^+^NK^+^, 1 as T^+^B^−^NK^−^, 3 patients with T^+^B^−^NK^+^ SCID (1 Omenn phenotype).

The median T cell counts/μL in typical SCIDs was significantly lower than leaky SCID (1[range, 0–388] vs. 1,165 [range, 493–8,288]; *p* < 0.0001) and CID (1,565 [range, 927–13,900]; *p* < 0.0001).

Though the median ALC/μL was significantly lower in the SCID group (including typical and leaky SCID) than CID (771 [11–9,570] counts/μL vs. 4,408 [2,860–1,9041] counts/μL; *p* = 0.0003), the median age of onset in the SCID group (46 [range, 0–730] days) was not statistically significant from the median age of onset within CID group (106 days [range, 15–304], *p* = 0.08).

Apart from lymphocyte subset analysis, serum immunoglobulin levels were measured in a total of 41 available patient serum samples. The median serum IgG level in all SCID subtypes was lower than the age matched ranges. However, 14 patients had normal Sr. IgG levels in our cohort. These included 6 cases of B^+^ SCID, 7 cases of B^−^ SCID, and 1 case of ZAP70 deficiency. Eight of these Fourteen patients were less than 6 months of age, suggesting the presence of maternal immunoglobulins in these children. Normal IgE was observed in 7 cases (1 case of B^+^ SCID, 2 cases of B^−^SCID, 4 patients with T^−^B^−^NK^+^) and elevated IgE was noted in 2 cases (T^−^B^+^ SCIDs).

Table 2 presents the basic immunological findings in our patient cohort.

**Table 2:** Immunological findings in SCID patients.

**Patient code**	**Genetic defect**	**WBC/μL**	**P(%)**	**L(%)**	**M(%)**	**E(%)**	**ALC/μl**	**B%**	**Abs counts/μL**	**T%**	**Abs counts/μL**	**Th%**	**Abs counts/μL**	**Tc%**	**Abs counts/μL**	**NK%**	**Abs counts/μL**	**IgG g/L**	**IgA g/L**	**IgM g/L**	**IgE IU/ml**
P1	*PNP*	38200	92	2	6	0	364	42	153	32	116	30	109	2	7	17	62	8.19	1.75	2.17	ND
P2	*RAG1 (OS)*	14870	19	21	1	59	3123	0	0	11	344	3	94	5	156	82	2561	1.41	0.249	.179	15.3
P3	*RFXAP*	14300	76	20	3	1	2860	59	1687	38	1087	8.5	243	29	829	2	57	ND	ND	ND	ND
P4	*ADA* (Leaky/atypical)	12850	84	6	10	0	771	3	23	85	655	34	262	38	293	11	85	2.64	0.56	0.3	28
P5	*ZAP70*	28850	23	66	5	6	19041	12	2285	73	13900	73	13900	0	0	15	2856	6.18	1.48	1.7	<4.4
P6	*RFXAP*	5700	54	39	0	7	3078	48	1477	48	1477	11	339	35	1077	3	92	ND	ND	ND	ND
P7	*IL2RG*	7020	32	43	12	13	3019	97	2928	1.7	51	0.7	21	1	30	1	30	2.7	0.2	0.04	5.3
P8	*IL2RG*	5750	42	48	10	0	2760	95	2622	0.1	3	0	0	0.1	3	3.2	88	ND	ND	ND	ND
P9	*IL2RG*	17810	72	15	10	3	2672	82	2191	0	0	0	0	0	0	12	321	1.54	0.235	0.302	735
P10	*IL2RG*	5390	81	14	6	ND	710	98	695.8	0	0	0	0	0	0	0	0	0.564	1.37	0.231	61.7
P11	*ADA*	12240	90	1	9	0	160	0	0	0	0	0	0	0	0	1	2	ND	ND	ND	ND
P12	*JAK3* (Leaky/atypical)	16370	50	32	6	13	5238	68	3562	26	1362	6	314	19	995	1	52	ND	ND	ND	ND
P13	*AK2*	570	4	44	33	0	251	29	73	0	1	0	0	0	0	41	103	7.37	0.343	0.92	ND
P14	*ADA*	11000	ND	0.1	ND	ND	11	0	0	0	0	0	0	0	0	0	0	ND	ND	ND	ND
P15	*JAK3*	34000	80	11	9	0	4752	95	4514	0	0	0	0	0	0	2	95	ND	ND	ND	ND
P16	*RAG1*	3530	52	36	7	5	1280	42	537.6	34	435.2	2	25.6	18	230.4	17	217.6	10.8	0.239	2.34	ND
P17	*ADA*	6490	90	1	8	0	80	0	0	0	0	0	0	0	0	100	80	1.41	0.32	0.12	ND
P18	*RAG2*	9980	93	1	4	2	91	0	0	1	1	0	0	0	0	98	89	0.226	0.239	0.168	<4.45
P19	– (Leaky/atypical)	10500	25	70	4	1	7350	13	956	42	3087	6	441	26	1911	8	588	3.1	1.08	1.97	ND
P20	*RFX5*	5750	10	62	26	2	3565	36	1283	26	927	6	214	16	570	36	1283	0.641	0.17	4.32	17.3
P21	*RFXAP*	5630	11	64	23	1	3610	43	1549	44	1585	19	685	19	685	9	324	ND	ND	ND	ND
P22	*ADA*	8220	38	10	39	13	822	0	0	0	0	0	0	0	0	0.1	13	ND	ND	ND	ND
P23	–	8300	90	2	7	0	130	4	7	2	3	0	0	0	0	89	148	ND	ND	ND	ND
P24	*RAG2*	7020	74	7.4	12	2.1	491	0	0	0.2	1	0.1	0	0.1	0	99	486	2.14	0.42	0.93	43.7
P25	*PRKDC (leaky)*	7900	72	17	11	0	1343	47	631	50	672	22	295	28	376	2	27	ND	ND	ND	ND
P26	*RAG1* (Leaky/atypical)	8910	72	19	9	0	1693	38	643	43	728	22	372	8	135	13	220	ND	ND	ND	ND
P27	–	20600	87	3	9	1	714	98	700	0.5	4	0.4	0	0	0	1	7	0.75	0.01	0.5	0.1
P28	–	7990	89	2	9	0	160	0.1	0	1	2	0.5	1	0.5	1	14	22	3.11	0.358	0.17	107
P29	*JAK3*	17720	94	5	2	0	886	93	823	0	0	0	0	0	0	0	0	0.13	0.06	0.14	ND
P30	–	1500	21	65	12	ND	315	0	0	0	0	0	0	0	0	100	315	1.69	0.239	0.168	17.8
P31	*IL7RA*	5420	61	20.3	17	0.7	1100	86	946	0	0	0	0	0	0		152	0.07	0.06	0.2	2
P32	*DCLER1C*	21470	73	10	15.2	2	1232	0	0	1	9	0.3	4	0.2	2	99	1220	1.4	0.06	0.04	2
P33	*CIITA*	11780	30	57	7	6	6750	75	5036	23	1544	8	537	13	873	1	47	0.0683	0.316	4.32	17.3
P34	–(Leaky/atypical)	6700	ND	41	ND	ND	2800	32	879	29	797	3	82	4	110	19	522	0.037	0.249	0.239	ND
P35	*RAG2* (Leaky/atypical)	4900	40	40	20	0	1940	1	18	65	1165	7	125	31	556	14	251	11.2	0.3	1.87	ND
P36	*JAK3*	5460	82	6	11	0.2	328	92	301	0	0	0	0	0	0	0	0	0.336	0.246	0.172	<4.45
P37	*IL2RG*	3610	44	14	38	3	510	90	455	0	0	0	0	0	0	0.5	3	3.96	0.246	0.172	<4.45
P38	*RAG1*	2440	46	22	31	0	537	0	0	0	0	0	0	0	0	90	483	ND	ND	ND	ND
P39	*JAK3* (OS)	19200	60	33	7	0	6330	35	2218	63	3992	11	697	53	3558	0	0	0.936	0.239	0.255	<4.45
P40	*JAK3*	41130	89	8	3	0	3250	99	3275	0	0	0	0	0	0	0	0	0.878	0.239	0.168	<4.45
P41	*IL2RG*	3470	88	7	1	4	240	98	238	1	2	1	2	0	0	0	0	0.33	0.45	0.06	ND
P42	*RAG1*	2500	96	3	1	0	75	0	0	0	0	0	0	0	0	99	74	ND	ND	ND	ND
P43	*RAG2* (OS)	13660	9	3	0	28	1250	0	ND	29	357	17	209	8	98	43	529	1.85	0.314	0.217	163
P44	*IL2RG (Leaky)*	7870	73	19	8	0	1495	65	972	33	493	8	120	24	359	1	15	12.9	0.843	2.07	976
P45	*IL2RG*	4580	73	15	12	ND	700	98	671	0	0	0	0	0	0	1	3	0.048	0.02	0.015	0
P46	*DCLER1C*	7930	62	9	29	0	710	0	0	0	0	0	0	0	0	99	709	0.0976	0.239	0.168	4.45
P47	–	11550	46	25	9	17	2930	39	1126	4	116	4	116	0	0	54	1559	ND	ND	ND	ND
P48	*RAG*	4550	93	5	1	1	210	0	0	0	0	0	0	0	0	3	6	ND	ND	ND	ND
P49	*RFXANK*	7770	4	67	23	5	5240	12	625	61	3176	34	1770	25	1301	26	1354	0.94	0.239	0.975	17.8
P50	*ZAP70 (Atypical)*	11240	27	56	15	2	6270	8	504	89	5602	42	2644	46	2895	1	63	2.2	0.6	0.216	ND
P51	*RAG1* (OS)	17400	38	55	7	0	9570	0	0	85	8288	ND	ND	ND	ND	8	780	0.179	0.001	0.08	19.8
P52	*JAK3*	16170	93	3	4	0.1	485	90	437	0	0	0	0	0	0	2.2	10	8.98	1.51	1.8	17.8
P53	*ADA*	3800	52	18	29	1	680	55	376	35	239	15	103	22	150	6	41	4.68	0.239	0.168	4.45
P54	*JAK3*	11500	ND	18	ND	ND	2403	99.6	2393	0.3	7	0	0	0	0	0	0	1.35	0.25	0.19	0.1
P55	*JAK3* (Leaky/atypical)	9270	47	41	11	0.4	3801	37	1406	53	2014	12	456	39	1482	9	342	6.91	0.359	1.61	17.8
P56	–	840	69	20	7	2	580	33	191	67	388	57	330	10	58	0	0	ND	ND	ND	ND
P57	*RAG1*	2630	69.2	10	14	6	263	1	3	18	47	16	42	3	8	80	210	8.29	0.239	2.1	56

*ND, no data; “–,”No mutation identified; OS, Omenn syndrome; WBC, White blood cell; P, polymorphonuclear neutrophils; L, lymphocytes; M, Monocytes; E, Eosinophils; B, basophils; Plt, Platelets; ALC/ul, absolute lymphocyte counts/uL; Th, T Helper cells; Tc, t cytotoxic cells; IgG, immunoglobulin G; IgA, immunoglobulin A; IgM, immunoglobulin M; IgE, immunoglobulin E; IL2RG, Interleukin 2 receptor gamma chain; JAK3, Janus Kinase 3; IL7RA, Interleukin 7 receptor alpha; ADA, Adenosine Deaminase; PNP, Purine nucleoside phosphorylase; AK2, adenylate kinase 2; RAG1/2, Recombination activating gene1/2; DCLER1C, DNA Cross-Link Repair 1C; RFXAP, Regulatory Factor X Associated Protein; RFX5, Regulatory factor 5; RFXANK, Regulatory Factor X Associated Ankyrin Containing Protein); CIITA, Class II Major Histocompatibility Complex Transactivator; ZAP70, Zeta-chain-associated protein kinase 70*.

Absent HLA-DR expression was noted in 6 of 57 patients and they were classified as MHC class II deficient cases. Elevated HLA-DR expression with other features of Omenn SCID such as skin rash, hepatosplenomegaly, elevated eosinophil counts was identified in 4 patients. T cell proliferation assay was performed in 3 leaky SCID patients and all had a poor T cell response to PHA.

The median percentage of naïve Th cells(0.3 [range, 0–8]%) within the typical and atypical SCID patient group was significantly lower than the median percentage of healthy age matched control group 73% ([range, 66–89]%; *p* < 0.0001).The median percentage of naïve Tc cells was significantly lower in SCID group than control group (7 [range, 0–29] vs. 64[range, 41–74]; *p* < 0.001). In MHC class II deficiency cases, a selective deficiency of naïve cells on CD4 cells was noted in 3 of 4 cases. The median percentage of naïve Th in MHC class II deficient cases was 39.50 (range, 29–48) which was significantly lower than the median percentage of healthy control group 77 ([range, 66–99]; *p* < 0.003). The naïve Tc percentage was within the normal ranges in these 3 cases. Measurement of naïve T cell percentages in one case of ZAP70 deficiency revealed reduction of naïve T cell subsets on both CD4 and CD8.

Four patients with T^−^B^−^NK^−^ and 1 patient with T^+^B^−^NK^−^ had RBC-ADA activity (≤0.5 U/g Hb) lower than the healthy controls (1.1–2.5 U/g Hb) and were sequenced for *ADA* gene defects.

Absent CD132 expression on B cells was observed in 6 patients and they were classified as X-SCID. Seven patients with B^+^ phenotype had detectable T cells and could be tested for JAK3-pSTAT5 signaling studies and CD127 expression. Of these, 2 patients (P12,39) had absent pSTAT5 expression on T cells after IL-2 stimulation and two patients (P12, P44) had reduced expression of CD127 on T cells.

Analysis of T cell receptor excision circles (TRECs) was done in all the SCID and CID patients and compared with TREC copies in age matched healthy controls samples (*n* = 55). The median TREC copies in T^−^B^+^ SCID (2.3[0.0175–16]) and T^−^B^−^ SCID (3[0–11]) was significantly lower than the control group (139 copies [range, 62–348]; *p* < 0.0001). The median TREC copies in MHC class II deficiency 81.5(13–154) and ZAP70 deficiency 40.5(17–64) were significantly higher than SCID patients (*p* < 0.01).

### Treatment and Outcome

Intravenous immunoglobulin was administered to 75% of the patients and 54% of patients were on prophylaxis (antibacterial, antiviral, and antifungal). During the study period, 4 patients (P6, P12, P13, P20) underwent HSCT however, had a poor survival outcome. The median age at transplant was 8 months (range, 2–12). P6 underwent umbilical cord blood transplant and died due to post-transplant complications like diarrhea and Gram-negative sepsis. P12 and P13 underwent haploidentical HSCT, however expired from Graft vs. Host disease and adenovirus infection, respectively. P20 underwent HSCT from HLA identical sibling, however, expired in the period immediately followed by HSCT due to lung damage and systemic candidiasis. P6 and P20 underwent HSCT using myeloablative conditioning (Treosulfan, Cyclophosphamide, anti-thymocyte globulin [ATG]). For the other two patients, no details were available on the conditioning regimen.

Of the remaining patients, 49 patients could be followed up. Presently, only 6 patients are surviving. These patients were recently diagnosed as SCID and the median age of these children is 5.5 months (range, 2–30). The patient aged 2.5 years (30 months) had a late onset of presentation (2 years). At the time of last available report, 2 of these 6 patients were awaiting a transplant.

The median age of death in patients who did not undergo HSCT (*n* = 43) was 6 months (range, 1.5 months−3 years). Majority of these children expired before 12 months of age (*n* = 38). Three patients survived beyond 1 year of age (Patient P1 with PNP deficiency, P5 with ZAP70 deficiency and P26 with hypomorphic *RAG* gene mutation). While P1 and P5 expired within 2 years of age, P26 expired at 3 years. The main cause of death in all the patients was respiratory failure, chronic diarrhea, sepsis and disseminated BCG in 1 patient.

### Molecular Findings

As a first-line approach, molecular investigations were done in the patients by sanger sequencing of the common genes like *IL2RG, IL7RA, ADA, RAG1, RAG2, ZAP70* gene depending on the immunophenotypic pattern. Twenty-five patients could be molecularly characterized using this approach. In a quest to identify the underlying genetic defect in the remaining cases, Targeted Next Generation sequencing (T-NGS)- Primary immunodeficiency (PID) panel was done (*n* = 32). Of the 32 cases referred for T-NGS, 24 cases could be molecularly characterized, however, 8 cases still remained uncharacterized.

Overall, a total of 49 patients could be molecularly characterized in this study. Of the 25 cases characterized by sanger sequencing, we identified 7 patients with mutations in *IL2RG*, 1 patient with *IL7Ra* deficiency, 5 with *ADA* deficiency, 1 with *PNP* defect, 1 with *AK2* defect,9 patients with *RAG1/2* deficiency and 1 case of *ZAP70* defect. With the help of T-NGS, genetic cause could be identified in 24 cases with 9 cases of *JAK3* deficiency (^*^1 case was identified with whole exome sequencing), 6 MHC class II deficient cases, 3 *RAG* 1/2mutations, 2 *IL2RG* defects, 1 *PRKDC* defect, 2 cases of *DCLER1C*, an atypical case of ZAP70 deficiency. The nature of novel missense mutations identified in our cohort was determined by *in silico* tools like Mutation Taster (10), SIFT (11) and Polyphen-2 (12). Depending on the availability of parent's sample, familial segregation analysis was performed.

The molecular findings are presented in Tables 3–7.

**Table 3:** Molecular findings in T^−/+^B^+^NK^−^SCID.

**Pt No**.	**Defective gene**	**Nucleotide change**	**Protein change**	**Mutation type**	**Allele**	**Carrier status**		**References**	**Method**
						**Mother**	**Father**		
P7	*IL2RG*	c.202 G>A	p. E68K	Missense	Hemizygous	Carrier	NA	(13)	Sanger
P8	*IL2RG*	c.202 G>A	p. E68K	Missense	Hemizygous	Carrier	NA	(13)	Sanger
P10	*IL2RG*	c.943 A>T	p. K315X	Nonsense	Hemizygous	Carrier	NA	This study	Sanger
P12	JAK3^*^	c.2072T>A	p. V691E	Missense	Homozygous	Carrier	Carrier	This study	NGS
P15	*JAK3*	c.2978G>A	p. W993X	Nonsense	Homozygous	Carrier	Carrier	This study	NGS
P25	PRKDC	c.9862C>T c.11588G>A	p. R3288W, p. R3863H	Missense Missense	Compound Heterozygous	ND	ND	This study	NGS
P29	*JAK3*	c.2488A>T	p. K830X	Nonsense	Homozygous	Carrier	Carrier	This study	NGS
P36	*JAK3*	c.595 C>T, c.2805^+^5G>A	p. R199C 5′proximal splice site	Missense, Splice site mutation	Compound Heterozygous	Carrier	Carrier	Reported This study	NGS
P37	*IL2RG*	c.676C>T	p. R226C	Missense	Hemizygous	ND	ND	(13)	NGS
P39	*JAK3*	c.862-2A>G, c.442 G>A	3′ Splice site Intron 6; p. G148R	Splice site Missense	Compound Heterozygous	Carrier	Carrier	This study, This study	NGS
P40	*JAK3*	c.2243delA	p. K748SfsTer8	Deletion	Homozygous	Carrier	Carrier	This study	NGS
P45	*IL2RG*	c.3 G>A	p. M1I	Frameshift	Hemizygous	ND	ND	(13)	Sanger
P31	*IL7RA*	c.437_438delTT	p. F146CfsTer5	Frameshift	Homozygous	Carrier	Carrier	This study	Sanger
P41	*IL2RG*	c.53delT	p. L18RfsTer6	Frameshift	Hemizygous	ND	ND	This study	Sanger
P44	*IL2RG*	c.979G>A	p. E327K	Missense	Hemizygous	Carrier	ND	This study	Sanger
P52	*JAK3*	c.2350 G>C	p. R784N	Missense	Homozygous	Carrier	Carrier	(14)	NGS
P54	*JAK3*	c.1351 C>T	p.R451X	Nonsense	Homozygous	Carrier	Carrier	This study	NGS
P55	*JAK3*	c.307 C>T c.421-6 C>T	p.R103C; 3′splice variant	Missense, Splice site	Compound Heterozygous	ND	ND	(15) This study	NGS

IL2RG, Interleukin 2 receptor gamma chain; JAK3, Janus Kinase 3; IL7RA, Interleukin 7 receptor alpha;

* *P12 was identified with the help of WES*.

**Table 4:** Molecular findings in T^−/+^B^+^NK^+^SCID.

**Pt No**.	**Defective gene**	**Nucleotide change**	**Protein change**	**Mutation type**	**Allele**	**Carrier Status**		**Reference**	**Method**
						**Mother**	**Father**		
P26	*RAG1*	c.2849delT	p. I950Mfster28	Deletion	Compound heterozygous	Carrier for c.2849delT	Carrier for c.1421G>A	This study	NGS
		c.1421G>A	p. R474H	Missense				(16)	
P9	*IL2RG*	c.749 del C	T250Ifster23	Frameshift	Hemizygous	NA	NA	This study	Sanger

*RAG1, recombination activating gene1; IL2RG, Interleukin 2 receptor gamma chain*.

**Table 5:** Molecular findings in T^−/+^B^−^NK^−^SCID.

**Pt No**.	**Defective gene**	**Nucleotide change**	**Protein change**	**Mutation type**	**Allele**	**Carrier status**		**References**	**Method**
						**Mother**	**Father**		
P1	*PNP*	c.199C>T	p.67R>X	Nonsense	Homozygous	Carrier	Carrier	This study	Sanger
P4	*ADA*	c.42 T>C	p. L14P	Missense	Homozygous	Carrier	Carrier	This study	Sanger
P11	*ADA*	c.523 C>T	p.Q175 X	Nonsense	Homozygous	Carrier	Carrier	This study	Sanger
		c.716 G>A	p. G239D	Missense	Homozygous	Carrier	Carrier	(17)	Sanger
P13	*AK2*	c.276 C>A	p.C92X	Nonsense	Homozygous	ND	ND	This study	Sanger
P14	*ADA*	c.3632A>G.	3′ splice variant	Splice site	Compound Heterozygous	Carrier	Carrier	This study	Sanger
		c.613_615del	p. Val205del	Deletion				This study	
P22	*ADA*	c.523 C>T c.716 G>A	p.Q175 X	Missense	Homozygous	Carrier	Carrier	This study,	Sanger
			p. G239D					(17)	
P53	*ADA*	c.716 G>A	p. G239D	Missense	Homozygous	Carrier	Carrier	(17)	Sanger
P48	*RAG1*	c.2146C>T	p. R716W	Missense	Homozygous	Carrier	Carrier	(16)	NGS
P17	IL2RG	c.331delA	p. I111SfsTer36	Frameshift	Hemizygous	Carrier	NA	This study	Sanger

*ADA, Adenosine Deaminase; PNP, Purine nucleoside phosphorylase; AK2, adenylate kinase 2*.

**Table 6:** Molecular findings in T^−/+^B^−^NK^+^SCID.

**Pt No**.	**Defective gene**	**Nucleotide change**	**Protein change**	**Mutation type**	**Allele**	**Carrier status**		**References**	**Method**
						**Mother**	**Father**		
P2	*RAG1*	c.994C>T	p. R331X	Nonsense	Homozygous	Carrier	Carrier	This study	Sanger
P18	*RAG2*	c.698G>T	p. G35V	Missense	Homozygous	Carrier	Carrier	(18)	Sanger
P24	*RAG2*	c.1321C>T	p. P441S	Missense	Homozygous	ND	Carrier	This study	Sanger
P32	*DCLRE1C*	c.879G>A	p. W293X	Nonsense	Homozygous	ND	ND	This study	Sanger
P35	*RAG2*	c.171delG,	p.K58STer73,	Frameshift	Compound Heterozygous	Carrier	Carrier	This study	Sanger
		c.104 G>C	p. G35A	Missense				(18)	
P38	*RAG1*	c.1201_1216del	p.S401LfsTer6	Frameshift	Homozygous	Carrier	Carrier	This study	Sanger
P42	*RAG1*	c.2146C>T	p. R716W	Missense	Homozygous	Carrier	Carrier	(16)	NGS
P43	*RAG2*	c.1247G>T	p.W416L	Missense	Homozygous	Carrier	Carrier	(19)	Sanger
P46	*DCLER1C*	Del Exon 1-3	NA	Deletion	Homozygous	ND	ND	(15)	NGS
P51	*RAG1*	c.437_438delAG	p.R147SfsTer21	Deletion	Homozygous	Carrier	Carrier	This study	NGS
P57	*RAG1*	c.619 G>A	p.W151X	Nonsense	Homozygous	ND	Carrier	This study	Sanger
P16	*RAG1*	c.1441 G>A	p.R474H	Missense	Compound Heterozygous	ND	ND	This study	NGS
		c.1442 G>A	P.T481C	Missense				(19)	

*RAG1/2, Recombination activating gene1/2; DCLER1C, DNA Cross-Link Repair 1C*.

**Table 7:** Molecular findings in CID.

**Pt No**.	**Defective gene**	**Nucleotide change**	**Protein change**	**Mutation type**	**Allele**	**Carrier status**		**References**	**Method**
						**Mother**	**Father**		
P3	*RFXAP*	c.460_461insC	p. K155QfsTer21	Frameshift	Homozygous	Carrier	Carrier	(20)	NGS
P6	*RFXAP*	c.460_461insC	p. K155QfsTer21	Frameshift	Homozygous	Carrier	Carrier	(20)	NGS
P20	*RFX5*	c.1154delT	L385YfsTer33	Frameshift	Homozygous	Carrier	Carrier	(20)	NGS
P21	*RFXAP*	c.709-1G>T	Intron 2	Splice site	Homozygous	Carrier	Carrier	(20)	NGS
P33	*CIITA*	c.2436C>A	p. C812X	Nonsense	Homozygous	ND	ND	(20)	NGS
P49	*RFXANK*	c.378_387del	p. P127GfsTer74	Frameshift	Homozygous	ND	ND	(20)	NGS
P5	*ZAP70*	c.183 T>A	p. RIle61N	Missense	Homozygous	Carrier	Carrier	(21)	Sanger
P50	*ZAP70*	c.847C>T	p. R283X	Nonsense	Homozygous	Carrier	Carrier	This study	NGS

*RFXAP, Regulatory Factor X Associated Protein; RFX5, Regulatory factor 5; RFXANK, Regulatory Factor X Associated Ankyrin Containing Protein); CIITA, Class II Major Histocompatibility Complex Transactivator; ZAP70, Zeta-chain-associated protein kinase 70*.

## Discussion

SCID is a genetically heterogenous group of disorders that affects both the cellular and humoral immunity. The incidence and prevalence of SCID varies in different parts of the world and is reported to be higher in countries with a high rate of consanguinity. To the best of our knowledge, this is the first comprehensive report on clinical, immunological, and molecular studies in 57 SCID patients from India.

Majority of our patients (89%) presented within 6 months of age with a median age of onset of 2 months. The median age of diagnosis in our cohort (152 days; 5 months) was consistent with other studies such as Canada (4.2 months), China (5 months), Greece (6 months), and United States (6.59 months) (22–24). Patients with MHC class II deficiency and ZAP70 deficiency were also referred to us within 1 year of age with a clinical suspicion of SCID. Though, these groups are categorized under “Combined immunodeficiency less profound than Severe combined immunodeficiency” our patient data highlights the clinical severity of these disorders to be like SCID.

We observed 36% rate of consanguinity in our cohort which is intermediate to countries like Iran, Kuwait (18) where a very high rate of consanguinity (77%) exists and UK which has 3% rate of consanguineous marriages (23).

Lymphopenia, which is considered as a hallmark of SCID was observed in 67% of our patients (cut off <2,500 counts/μL). The patients with normal ALC/μl included B^+^ SCIDs (*n* = 10) and Omenn SCID (*n* = 4). MHC class II deficient patients and ZAP70 deficient patients also had normal ALC/μl. This is consistent with a study from china where 86% of the patients had a low lymphocyte count (25). Pilot series from United States identified most newborns with SCID based on ALC, but, 10% of SCID samples had normal lymphocyte counts.

The median TREC copies/μL were significantly lower in SCID patients as compared to healthy controls. Importantly, we found TREC copies to be higher than typical SCIDs in children with either CD4 or CD8 lymphopenia. Both TREC copies and ALC/μL are higher in MHC class II deficiency and ZAP70 deficiency than typical SCIDs. Hence, in a scenario of severe infections with normal ALC/μL and normal TRECs, extensive immunological evaluations should still be performed to rule out these forms of SCID. An important clue to underlying immunodeficiency in both these cases were reduced percentages of naïve Th/Tc which was evaluated in 4 cases of MHC class II deficiency and 1 case of ZAP70 deficiency. Notably, 3 of 4 MHC class II deficiency cases showed a selective reduction of Naïve Th population with normal percentages of naive Tc cells. Hence, a suspicion of MHC class II deficiency can be made in a scenario of selective reduction of naïve Th subset.

Traditionally SCID patients have been classified based on absolute counts of T, B, NK cells. Majority of our cases belonged to the category of T^−^B^−^ SCID (39%) followed by T^−^B^+^ SCID (28%). In Greece, 40% SCID patients belonged to T^−^B^−^NK^+^ category and in Serbia and Montenegro, 57% patients with SCID and Omenn syndrome presented with T^−^B^−^NK^+^ SCID phenotype (26). In the registry of Saudi Arabia for combined immunodeficiencies, T^−^B^−^ was the most common type; 17% followed by T^−^B^+^ found in 5% and Omenn syndrome in 3.6% (26). China's SCID registry has reported 66.7% of patients with B^+^ SCID and 7.1% of the cohort with B^−^ SCID (25). Defective expression of major histocompatibility complex class II (MHC) molecules accounted for 5% of severe combined immunodeficiency (SCID) in Canadian Survey and almost 20–30% of SCID cases in Kuwait and North Africa (27). MHC class II deficiency accounted for 10.5%and ZAP70 deficiency constituted 3.5% of our patient group.

The pattern of lymphocyte subsets serves as a useful guide to perform genetic studies, however, molecular diagnosis of SCID is highly challenging due to the involvement of multiple genes whose defect can result in same immunophenotypic pattern. Hence, in such cases DNA sequencing of individual genes becomes tedious, time consuming and expensive. As there exists a significant clinical and immunophenotypic overlap between different genetic subtypes of SCID, we performed assays like measurement of RBC-ADA levels, flow cytometric evaluation of HLA-DR, CD132, CD127, and pSTAT5 expression to narrow down the list of possible genetic defects.

Spectrophotometric estimation of RBC-ADA levels was found to be a simple, cost- effective and accurate method for identification of ADA deficient patients. Five patients had low RBC-ADA levels and were also detected with a molecular defect in the *ADA* gene. Of the remaining patients with T^−^B^−^NK^−^ phenotype (*n* = 6), a *PNP* and *AK2* defect were identified. No pathogenic variant was identified in 2 patients despite T-NGS. In the remaining two patients, mutations were identified in *IL2RG* and *RAG1* gene. These findings expand the phenotypic spectrum of typical X-SCID and RAG SCID.

Study of HLA-DR expression on immune cells helped in identification of MHC class II deficient patients (absent HLA-DR expression) and Omenn SCID (elevated HLA-DR expression suggesting activated T lymphocytes).

Absent/reduced expression of CD132 on B cells helped identify 6 patients (67%) with *IL2RG* gene defect. One patient with c.676 C>T in Exon 5 of *IL2RG* had a normal expression of CD132 on B cells (89%) suggesting this mutation did not affect the protein expression. Functional screening of STAT3 phosphorylation after IL-21 stimulation to assess the functionality of γchain could have helped us in this scenario, but this assay was not performed in our study.

Two patients with T^+^B^+^ phenotype were identified with *RAG1/2* and *PRKDC* gene defect suggesting these mutations to be hypomorphic thereby, producing residual number of T and B cells. The oligoclonality of TCR-Vβ repertoire was tested in the RAG deficient patient and was found to be clonally restricted (Vβ13.1 on CD4^+^T cells [20.5%] and Vβ11[5%], Vβ16 [4.1%] on CD8^+^T cells). A case of *IL7RA* gene defect was detected in a patient with T^−^B^+^NK^−^ phenotype. CD127 expression studies could not be performed in the patient due to lack of T cells.

We observed reduced CD127 expression in 2 patients (P12 and P44) who were later identified with a mutation in *JAK3* gene and *IL2RG* gene, respectively. The possible explanation for this observation lies in the mechanism of IL7Ra receptor downregulation/ internalization due to high circulating levels of IL7 in some lymphopenic patients. However, studies to look for internalized CD127 or western blot from separated T cells to look for CD127 expression could not be performed in our study. Hence, a reduced CD127 expression on flow cytometry needs to be interpreted carefully.

A hemizygous deletion (c.749 del C) in Exon 5 of *IL2RG* gene leading to frameshift mutation was identified in a T^−^B^+^NK^+^ patient (male). This patient had a normal CD132 expression on B cells, but its functionality was not tested. Normal NK cell numbers in γchain deficient patients have been reported earlier (28) and they are predicted to be of maternal origin. In patients with mutations involving the γc portion of the *IL2RG*, normal NK cell numbers have been identified and these observations have raised the possibility of a potential downstream activation mechanism in NK cell differentiation (28).

Flow cytometric evaluation of proteins specifically expressed on T cells (for e.g.,: CD127) or assays that required TCR engagement with specific stimulants (JAK3-pSTAT5) could not be assessed in patients who lacked T cells. Almost 65% of our B^+^ SCID cohort had absent T cells hence, both CD127 and phospho-STAT5 assay had a limited utility in our study and we had to rely on genetic analysis to identify the defect.

The frequency of mutations in *RAG1/2* (21 %) in the current study is like United States (21%) and the Netherlands (32%), but much less compared to Greece (41%) and Serbia (61%) where a common founder gene defect in RAG1 is likely (18). A patient with microcephaly, flat nasal bridge, short philtrum was suspected of a defect in DNA Ligase IV (1) but was identified with a *RAG1* defect. Three patients within this group were classified as Omenn SCID (2 *RAG1* mutations and 1 *RAG2* mutation).

The molecular findings in five cases of MHC class II deficiency from our cohort has been recently described (20). Additionally, we have identified another patient with a defect in *RFXANK* gene. There have been only 2 patients with confirmed *RFXANK* mutations reported in Asia. *RFXANK* mutation causes bare lymphocyte syndrome type 2B (15) commonly observed in North Africa (15) and in other places such as France and Spain.

An atypical case of ZAP70 deficiency with a novel nonsense mutation in *ZAP70* gene was identified in 1 patient with elevated CD8^+^T cells. Poor proliferative responses was the only clue to an underlying immunodeficiency in this patient. Unfortunately, we could not assess the intracellular ZAP70 expression or assess the TCR-Vβ repertoire on CD4 and CD8 T cells as the child expired by the time of receiving a molecular diagnosis. This mutation further extends the phenotypic spectrum of ZAP70 deficiency.

The spectrum of genetic defects in our cohort revealed a wide genetic heterogeneity with 21% *RAG1/2* defects,15.8% *IL2RG* defects, 15.8% *JAK3* defect, 1.7% *IL7RA* defect, 8.8% *ADA* defect, 1.8% *AK2* defect, 1.8% *PNP* defect, 3.5% *DCLER1C* defect, 1.8% *PRKDC* defect, 10.5% with MHC class II deficiency and 3.5% *ZAP70* defects. Thirty-two novel variants were identified in our study and no founder mutation was detected in our cohort. 14% of the defects still remained uncharacterized despite application of T-NGS (list of genes covered in the T-NGS panel is presented in Table 8) and it would be interesting to perform whole exome/genome sequencing in these cases as that may lead to discovery of novel genetic defects causing SCID (no pathogenic variant explaining the cause of immunodeficiency could be identified in one patient [P47] even after WES). Our data also shows a lack of demonstrable correlation between genotype and phenotype in few cases (7%). Certain genetic and environmental factors, concurrent mutations in other SCID genes, modifier gene(s), and mutations, that lead to sparing or disrupting developments of other lineages of lymphocytes may be the cause for lack of correlation (15).

**Table 8:** Genes covered in Targeted NGS panel.

*ACP5*	*ACTB*	*ADA*	*IFNAR2*	*IFNGR1*	*IFNGR2*	*RNASEH2A*	*RNASEH2C*	*TRAC*
*ADAM17*	*ADAR*	*AICDA*	*IGHM*	*IGKC*	*IGLL1*	*RNF168*	*RNU4ATAC*	*TREX1*
*AIRE*	*AK2*	*AP1S3*	*IKBKB*	*IKZF1*	*IL10*	*RORC*	*RTEL1*	*TTC7A*
*AP3B1*	*AP3D1*	*APOL1*	*IL10RA*	*IL10RB*	*IL12B*	*SAMD9*	*SAMHD1*	*UNC93B1*
*ATM*	*ATP6AP1*	*B2M*	*IL12RB1*	*IL17F*	*IL17RA*	*SBDS*	*SERPING1*	*USP18*
*BCL10*	*BCL11B*	*BLM*	*IL17RC*	*IL1RN*	*IL21R*	*SH2D1A*	*SLC29A3*	*WAS*
*BLNK*	*BTK*	*C1QA*	*IL2RA*	*IL2RG*	*IL36RN*	*SLC35C1*	*SLC46A1*	*WRAP53*
*C1QB*	*C1QC*	*C1R*	*IL7R*	*INO80*	*IRAK1*	*SMARCAL1*	*SNX10*	*ZBTB24*
*C1S*	*C2*	*C3*	*IRAK4*	*IRF2BP2*	*IRF3*	*SP110*	*STAT1*	
*C5*	*C6*	*C7*	*IRF7*	*IRF8*	*ISG15*	*STAT2*	*STAT5B*	
*C8A*	*C8B*	*C8G*	*ITCH*	*ITGB2*	*ITK*	*STIM1*	*STK4*	
*C9*	*CARD11*	*CARD14*	*JAGN1*	*JAK1*	*JAK3*	*STXBP2*	*STX11*	
*CARD9*	*CASP10*	*CASP8*	*KDM6A*	*KMT2D*	*LAMTOR2*	*TAPBP*	*TAP2*	
*CCBE1*	*CD19*	*CD247*	*LAT*	*LCK*	*LIG1*	*TBX1*	*TBK1*	
*CD27*	*CD3D*	*CD3E*	*LIG4*	*LPIN2*	*LRBA*	*TCN2*	*TCIRG1*	
*CD3G*	*CD40*	*CD40LG*	*LYST*	*MAGT1*	*MALT1*	*TFRC*	*TERT*	
*CD46*	*CD55*	*CD59*	*MAP3K14*	*MASP2*	*MCM4*	*TINF2*	*TICAM1*	
*CD79A*	*CD79B*	*CD81*	*MEFV*	*MKL1*	*MOGS*	*TMC6*	*TLR3*	
*CD8A*	*CDCA7*	*CEBPE*	*MS4A1*	*MSH6*	*MSN*	*TNFAIP3*	*TMEM173*	
*CECR1*	*CFB*	*CFD*	*MTHFD1*	*MVK*	*MYD88*	*TNFRSF13C*	*TNFRSF13B*	
*CFH*	*CFHR1*	*CFHR2*	*MYSM1*	*NBAS*	*NBN*	*TNFSF11*	*TNFRSF4*	
*CFHR3*	*CFHR4*	*CFHR5*	*NCF2*	*NCF4*	*NCSTN*	*TPP2*	*TPP1*	
*CFI*	*CFP*	*CFTR*	*NDNL2*	*NFAT5*	*NFKB1*	*TRAF3IP2*	*TRAF3*	
*CHD7*	*CIITA*	*CLCN7*	*NFKB2*	*NFKBIA*	*NHEJ1*	*TTC37*	*TRNT1*	
*CLPB*	*COPA*	*CORO1A*	*NHP2*	*NLRC4*	*NLRP1*	*UNC13D*	*TYK2*	
*CR2*	*CSF2RA*	*CSF2RB*	*NLRP12*	*NLRP3*	*NOD2*	*USB1*	*UNG*	
*CSF3R*	*CTC1*	*CTLA4*	*NOP10*	*OBFC1*	*ORAI1*	*VPS45*	*VPS13B*	
*CTPS1*	*CTSC*	*CXCR4*	*OSTM1*	*OTULIN*	*PARN*	*WIPF1*	*WDR1*	
*CYBA*	*CYBB*	*DCLRE1C*	*PEPD*	*PGM3*	*PIK3CD*	*ZAP70*	*XIAP*	
*DDX58*	*DKC1*	*DNAJC21*	*PIK3R1*	*PLCG2*	*PLEKHM1*	*RNASEH2B*	*TAP1*	
*DNMT3B*	*DOCK2*	*DOCK8*	*PMS2*	*PNP*	*POLA1*	*RNF31*	*TAZ*	
*ELANE*	*EPG5*	*ERCC6L2*	*POLE*	*POLE2*	*PRF1*	*RPSA*	*TCF3*	
*EXTL3*	*FAAP24*	*FADD*	*PRKCD*	*PRKDC*	*PSEN1*	*SAMD9L*	*TERC*	
*FAS*	*FASLG*	*FAT4*	*PSENEN*	*PSMB8*	*PSTPIP1*	*SEMA3E*	*THBD*	
*FCGR3A*	*FCN3*	*FERMT3*	*PTEN*	*PTPRC*	*RAB27A*	*SH3BP2*	*TIRAP*	
*FOXN1*	*FOXP3*	*FPR1*	*RAC2*	*RAG1*	*RAG2*	*SLC37A4*	*TMC8*	
*G6PC3*	*G6PD*	*GATA2*	*RANBP2*	*RASGRP1*	*RBCK1*	*SMARCD2*	*TNFRSF11A*	
*GFI1*	*HAX1*	*HELLS*	*RFX5*	*RFXANK*	*RFXAP*	*SPINK5*	*TNFRSF1A*	
*HMOX1*	*ICOS*	*IFIH1*	*RHOH*	*RLTPR*	*RMRP*	*STAT3*	*TNFSF12*	

The autosomal recessive form of SCID (86%) was more common than X-SCID (14%) in our cohort. This is in contrast with several studies that reported a predominance of X-linked SCID which accounts for approximately half of SCID cases (24). This finding supports that genetic defects in SCID patients probably differ depending on diverse genetic backgrounds. Our data is similar to countries where consanguinity is practiced like in Turkey where the AR form accounts for 80% SCID cases (29) as compared to data from USA with AR form 20% and a low rate of consanguinity (23).

The overall outcome in our cohort is extremely poor with death occurring in 92% of the patients. Only 4 patients underwent HSCT, and none of them could survive the transplant. The median age at HSCT in our cohort was 7.5 months. It is well known that HSCT for (S)CID before the age of 3.5 months results in a superior outcome (6). One of our patients with MHC class II deficiency (P7) had a pre-symptomatic diagnosis and received an early transplant (at 3 months), however, he expired within 8 days of transplant due to severe diarrhea and gram-negative sepsis. As stated in a recent report, the reason for low survival rate in MHC class II deficiency patients may be due to presentation of donor antigens by donor antigen-presenting cells to recipient T cells leading to graft rejection (30).

We consider delayed diagnosis as the major cause for a poor survival outcome in our cohort. Our data is consistent with countries where newborn screening (NBS) for SCID has not yet been implemented (18). Without NBS, asymptomatic diagnosis for SCID is possible only in a scenario of strong family history. In several other cases, even before the child is diagnosed as SCID and the crucial decision for transplantation is taken, the child has significant number of infections which affects the survival outcome. Maintenance on IVIg therapy is expensive and a major constraint in the management of children with PIDs in India, as also in other developing countries (31). Only two state governments (Punjab and Karnataka) in India have taken a major policy initiative to provide IVIg freely to the patients. In the other states, several children are not privileged to get the recommended dose of IVIg (31). Many centers in India are now routinely performing HSCT for a variety of malignant diseases in both children and adults whose results are comparable to many international published reports (32), however, there is only a minority that have the requisite expertise for carrying out transplant for PIDs (31). A recent report by a tertiary referral center in India shared their 15-year experience of HSCT in children with PIDs with encouraging results of an overall survival rate of 62 and 55% survival rate for SCIDs (33). However, the number of such centers that specialize in transplantation for PIDs in India are very few and specific arrangements are needed to transfer the patients to centers in other states. Financial constraint is one of the major reasons for families to refuse transplantation. Overall, delayed diagnosis due to lack of awareness about SCID among the pediatricians, lack of expertise in HSCT for SCID, financial constraints and lack of a suitable donor are the reasons for such a poor survival rate observed in our cohort.

Prenatal diagnosis (PND) is hence, highly useful in families affected with SCID. The preferred procedure for prenatal diagnosis is genetic confirmation in the index case and parents and then performing PND by chorionic villus sampling or amniocentesis. This was helpful in two of our affected families (index case P5 and P6). Two families (index case P6 and P42) benefited from the facility of phenotypic prenatal diagnosis performed on cordocentesis sample at 18 weeks of gestation due to lack of genetic diagnosis at the time of PND.

Early detection of SCID and HSCT at a pre-symptomatic stage has proven to result in a better outcome in countries with NBS program for SCID and advanced health care systems. As NBS for SCID is not yet performed in our country, there exists a high chance that we are missing out many cases as these children may have expired before receiving a diagnosis. Pilot studies for NBS in our country could provide data on the true incidence of SCID in our population. Overall, our data reveals a wide genetic heterogeneity of SCID in the Indian population, confirms the poor prognosis of SCID due to delayed diagnosis and highlights the need for implementing NBS for SCID in India.

## Author Contributions

JA analyzed the data and wrote the manuscript. MG, AD, SM, and MKu were involved in performing laboratory investigations of the different cases. MD, AT, NS, AA, HL, VK, GG, and MKa supervised the clinical care of the various patients. UB helped in procuring the clinical details and follow up of the patients. SR, JN, and JS provided the WES support. VT provided genetic diagnosis in *AK2* deficient patient. MM supervised the study and reviewed the manuscript.

### Conflict of Interest Statement

The authors declare that the research was conducted in the absence of any commercial or financial relationships that could be construed as a potential conflict of interest.
